# Supplementary figures and images for: Brain circuits activated by female sexual behavior evaluated by manganese enhanced magnetic resonance imaging
Source: PLoS One. 2022 Aug 1;17(8):e0272271. doi: 10.1371/journal.pone.0272271 (PMC9342731; doi:10.1371/journal.pone.0272271)

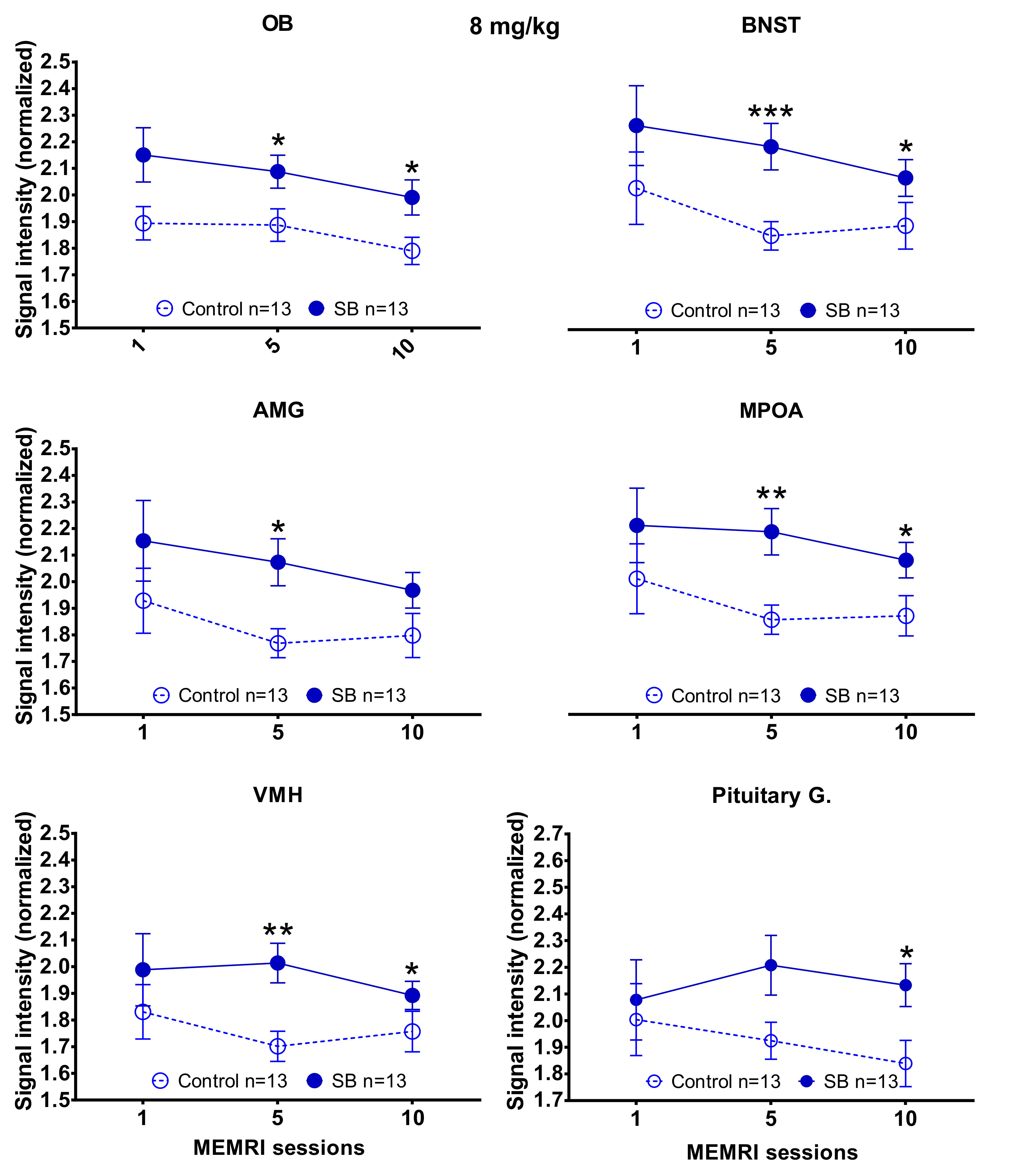

Supplement: S1 Fig — Data are expressed as mean ± SEM. * Different from control p<0.05; ** p<0.01; *** p<0.0001. (TIF) [file pone.0272271.s001.tif]

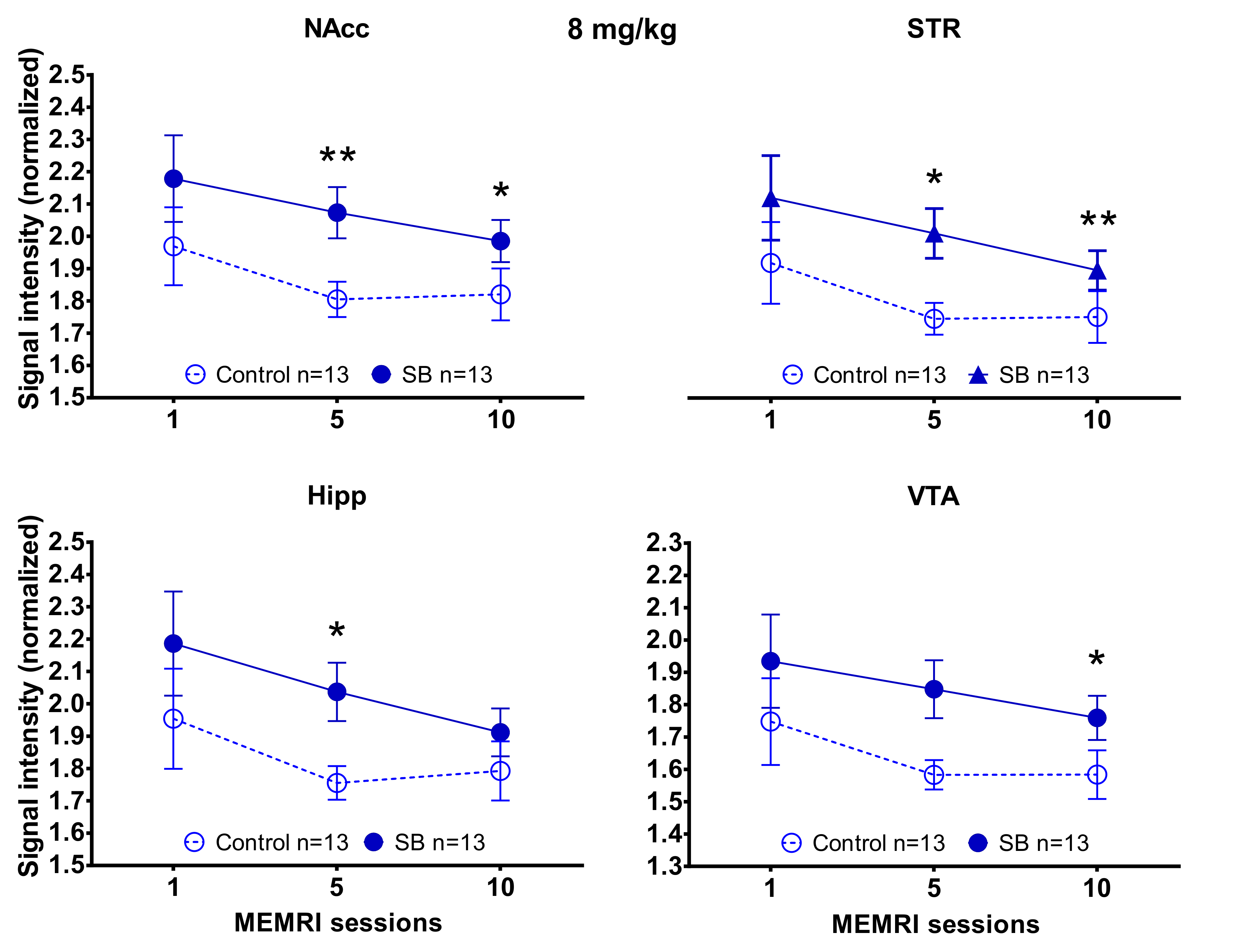

Supplement: S2 Fig — Data are expressed as mean ± SEM. * Different from control, p<0.05; **, p<0.01. (TIF) [file pone.0272271.s002.tif]

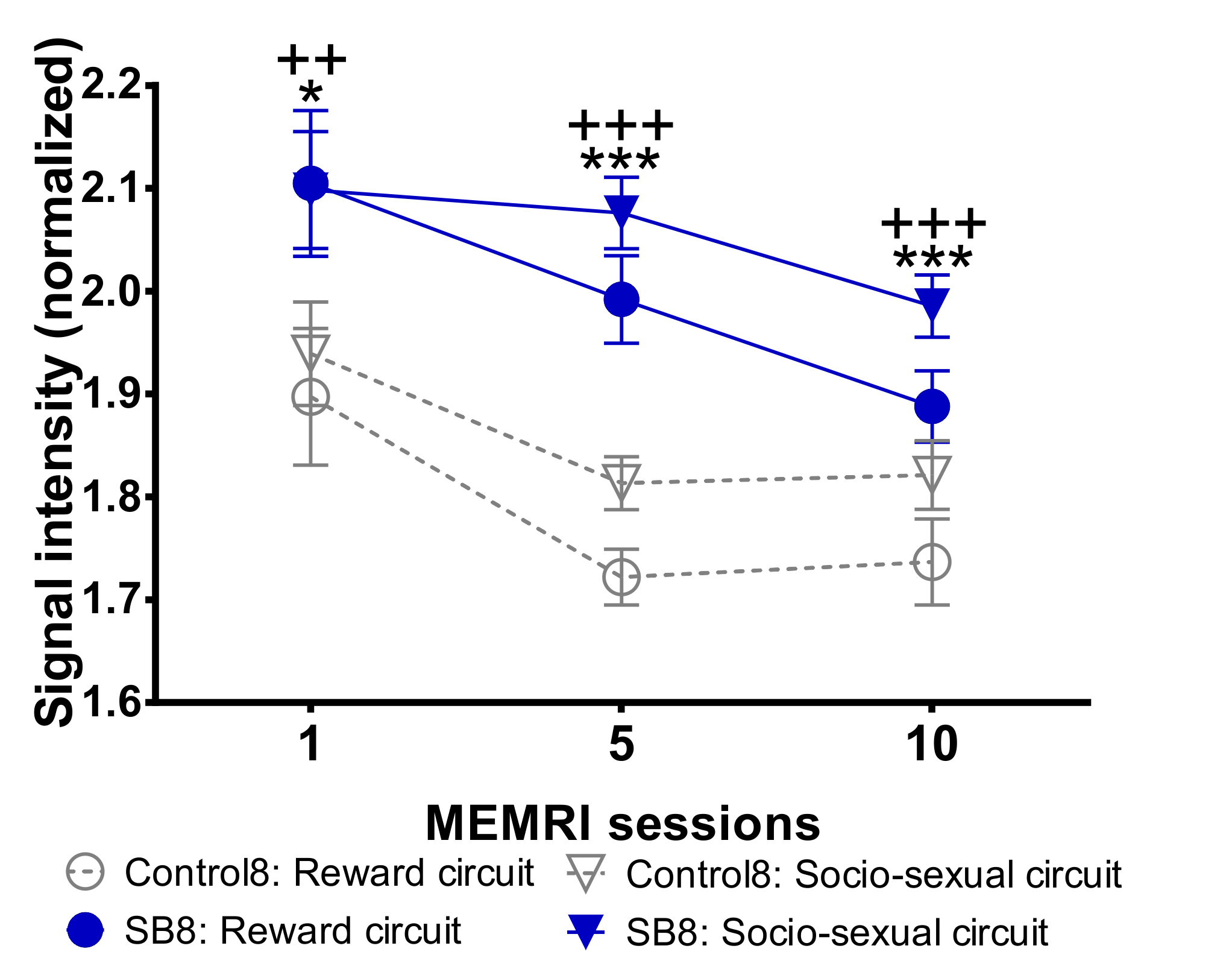

Supplement: S3 Fig — Data are expressed as mean ± SEM. * Socio-sexual circuit, different from control in the same session p<0.05; **, p<0.01; ***, p<0.001. ++ Reward circuit different form control in the same session, p<0.01; +++ p <0.001. (TIF) [file pone.0272271.s003.tif]
