# Supplementary material for: Brain circuits activated by female sexual behavior evaluated by manganese enhanced magnetic resonance imaging
Source: PLoS One. 2022 Aug 1;17(8):e0272271. doi: 10.1371/journal.pone.0272271 (PMC9342731; doi:10.1371/journal.pone.0272271)
Supplement: S1 Table — (DOCX) [file pone.0272271.s004.docx]

| **Supplementary Table 1.** Statistical results using the Kruskal Wallis and the Friedman repeated measures test on the sexual behavior parameters comparing the different groups in experiment 1. | | | | | | | |
| --- | --- | --- | --- | --- | --- | --- | --- |
|  | **Kruskal Wallis** | | | | **Friedman** | | |
|  | S1 | S5 | S10 | Control | | 8 mg/kg | 16 mg/kg |
| Mounts | H=0.9, p=0.6 | H=4.2, p=0.121 | H=2.7, p=0.257 | X^2^=4.32 p=0.12 | | X^2^=1.867, p=0.531 | X^2^=5.1, p=0.1 |
| Intromissions | H=0.4, p=0.8 | H=0.73, p=0.69 | H=3.97, p=0.137 | X^2^=2.6, p=0.285 | | X^2^=6.0 p=0.047 | X^2^=0.2, p=0.9 |
| Ejaculations | H=2.4, p=0.3 | H=0.02, p=0.98 | H=3.78, p=0.151 | X^2^=0.4, p=0.967 | | X^2^=3.556 p=0.285 | X^2^=2.8, p=0.3 |
| **Latencies (sec)** |  |  |  |  | |  |  |
| Mounts | H=0.3, p=0.8 | H=2.53, p=0.28 | H=2.98, p=0.22 | X^2^=0.45, p=0.79 | | X^2^=1.750, p=0.531 | X^2^=2.5, p=0.3 |
| Intromissions | H=0.3 p=0.9 | H=0.42, p=0.81 | H=0.46, p=0.792 | X^2^=1.75, p=0.53 | | X^2^=1.750, p=0531 | X^2^=3 p=0.28 |
| Ejaculations | H=0.1, p=0.9 | H=5.58 p=0.06 | H=0.603 p=0.74 | X^2^=0.81, p=0.79 | | X^2^=0.828, p=0.794 | X^2^=4 p=0.15 |
| **III (sec)** | H=0.6, p=0.7 | H=2.83, p=0.24 | H=0.61, p=0.734 | X^2^=1.18, p=0.65 | | X^2^=0.83, p=0.794 | X^2^=2.8, p=0.3 |
| **MLI** | H=3.4, p=0.1 | H=1.05, p=0.59 | H=0.0, p=1.0 | X^2^=2, p=0.794 | | X^2^=2, p=0.794 | X^2^=2, p=0.65 |
| **LQ** | H=2, p=0.36 | H=2.0, p=368 | H=0.0, p=1.0 | X^2^=2.0, p=0.794 | | X^2^=4.0, p=0.794 | X^2^=2, p=0.79 |
| **Return latencies after (sec)** |  |  |  |  | |  |  |
| Mounts | H=0.1, p=0.9 | H=2.53, p=0.28 | H=0.37, p=0.828 | X^2^=1.81, p=0.53 | | X^2^=2.769, p=0.355 | X^2^=3.9, p=0.2 |
| Intromissions | H=0.3, p=0.9 | H=1, p=0.595 | H=0.22, p=0.896 | X^2^=1.75, p=0.53 | | X^2^=1.750, p=0.53 | X^2^=1.7, p=0.5 |
| Ejaculations | H=2.5, p=0.3 | H=0.25, p=0.88 | H=1.66, p=0.435 | X^2^=0.63, p=0.79 | | X^2^=0.929, p=0.654 | X^2^=2.5, p=0.3 |
| **Percentage of exits after** |  |  |  |  | |  |  |
| Mounts | H=1, p=0.6 | H=3.1, p=0.216 | H=0.238, p=0.88 | X^2^=0.85, p=0.79 | | X^2^=4.0, p=0.236 | X^2^=2.5, p=0.3 |
| Intromissions | H=5.2, p=0.1 | H=0.16, p=0.92 | H=1.126, p=0.57 | X^2^=1.35, p=0.53 | | X^2^=1.0, p=0.654 | X^2^=4.8, p=0.1 |
| Ejaculations | H=1.3, p=0.5 | H=2.7, p=0.258 | H=2.25, p=0.324 | X^2^=0.66, p=0.96 | | X^2^=2.0, p=0.531 | X^2^=4.3, p=0.3 |
