# Supplementary material for: Brain circuits activated by female sexual behavior evaluated by manganese enhanced magnetic resonance imaging
Source: PLoS One. 2022 Aug 1;17(8):e0272271. doi: 10.1371/journal.pone.0272271 (PMC9342731; doi:10.1371/journal.pone.0272271)
Supplement: S2 Table — a) Statistical results of the Kruskal Wallis test for the running wheel and rotarod (in both modalities) comparing the different groups in experiment 1.b). Statistical results comparing the running wheel and rotarod (in both modalities) using the Friedman repeated measures test in experiment 1. (DOCX) [file pone.0272271.s005.docx]

| **Supplementary Table 2a.** Statistical results of the Kruskal Wallis test for the running wheel and rotarod (in both modalities) comparing the different groups in experiment 1. | | | | | | | | | | | | | |
| --- | --- | --- | --- | --- | --- | --- | --- | --- | --- | --- | --- | --- | --- |
|  | S1 | S2 | | S3 | S4 | S5 | | S6 | S7 | S8 | | S9 | S10 |
| Running wheel | H=2.085, p=0.353 | H=0.815, p=0.665 | | H=5.781, p=0.056 | H=5.288, p=0.071 | H=0.725, p=0.696 | | H=5.985, p=0.05 | H=5.554, p=0.062 | H=6.140, p=0.046 | | H=10.905, p=0.004 | H=0.62, p=0.733 |
|  |  |  | |  |  |  | |  |  |  | |  |  |
| Rotarod 10 rpm | H=2.246, p=0.325 | H=1.147, p=0.564 | | H=1.63, p=0.653 | H=1.049, p=0.592 | H=5.471, p=0.065 | | H=0.278, p=87 | H=0.837, p=0.658 | H=2.584, p=0.275 | | H=5.43, p=0.066 | H=0.138, p=0.933 |
|  |  |  | |  |  |  | |  |  |  | |  |  |
| Rotarod 10-15 rpm | H=1.898, p=0.387 | H=1.222, p=543 | | H=3.439, p=0.179 | H=2.46, p=0.292 | H=2.754, p=0.252 | | H=0.0498, p=0.975 | H=2.408, p=0.3 | H=5.269, p=0.072 | | H=2.494, p=0.287 | H=0.386, p=0.825 |
|  |  |  | |  |  |  | |  |  |  | |  |  |
| **Supplementary Table 2b.** Statistical results comparing the running wheel and rotarod (in both modalities) using the Friedman repeated measures test in experiment 1. | | | | | | | | | | | | | |
|  | | | Control | | | | 8 mg/kg | | | | 16 mg/kg | | |
| Running wheel | | | X^2^=9.335, p=0.407 | | | | X^2^=17.344, p=0.044 | | | | X^2^=26.55, p=0.002 | | |
| Rotarod 10 rpm | | | X^2^=14.303, p=0.112 | | | | X^2^=13.002, p=0.163 | | | | X^2^=14.992, p=0.091 | | |
| Rotarod 10-15 rpm | | | X^2^=5.158, p=0.82 | | | | X^2^=13.452, p=0.143 | | | | X^2^=7.104, p=0.626 | | |
