# Supplementary material for: Brain circuits activated by female sexual behavior evaluated by manganese enhanced magnetic resonance imaging
Source: PLoS One. 2022 Aug 1;17(8):e0272271. doi: 10.1371/journal.pone.0272271 (PMC9342731; doi:10.1371/journal.pone.0272271)
Supplement: S3 Table — (DOCX) [file pone.0272271.s006.docx]

| **Supplementary Table 3.** Statistical results of the Mann Whitney and the Friedman repeated measures ANOVA test on parameters comparing the different groups in experiment 2. | | | | | |
| --- | --- | --- | --- | --- | --- |
|  | **Mann Whitney** | | | **Friedman** | |
|  | S1 | S5 | S10 | 8 mg/kg | 16 mg/kg |
| Mounts | MWU=81, T=179, p=0.87 | MWU=73, T=187, p=0.57 | MWU=68, T=159, p=0.4 | X^2^=4.776, p=0.092 | X^2^=2.792, p=0.248 |
| Intromissions | MWU=81, T=178.5, p=0.89 | MWU=80, T=171, p=0.84 | MWU=66, T=193, p=0.36 | X^2^=1.846, p=0.397 | X^2^=2.28, p=0.32 |
| Ejaculations | MWU=78, T=182, p=0.736 | MWU=73, T=186, p=0.6 | MWU=82, T=178, p=0.91 | X^2^=8.977, p=0.011 | X^2^=6.195, p=0.045 |
| **Latencies (sec)** |  |  |  |  |  |
| Mounts | MWU=65, T=156.5, p=0.34 | MWU=46, T=137, p=0.05 | MWU=38, T=129, p=0.02 | X^2^=6.157, p=0.046 | X^2^=0.615, p=0.735 |
| Intromissions | MWU=63, T=154.5, p=0.29 | MWU=49, T=140, p=0.07 | MWU=49.5, T=140.5, p=0.07 | X^2^=5.692, p=0.058 | X^2^=2.462, p=0.292 |
| Ejaculations | MWU=84, T=175, p=1 | MWU=74, T=165, p=0.6 | MWU=83.5, T=176.5, p=0.98 | X^2^=2.923, p=0.897 | X^2^=0.542, p=0.763 |
| **III (sec)** | MWU=84, T=175, p=1 | MWU=82, T=178, p=0.92 | MWU=83.5, T=176.5, p=0.98 | X^2^=2.923, p=0.232 | X^2^=2.375, p=0.305 |
| **MLI** | MWU=78.5, T=169, p=0.6 | MWU=71, T=162, p=0.16 | MWU=84, T=175, p=1 | X^2^=0.0, p=1 | X^2^=2, p=0.368 |
| **LQ** | MWU=84, T=175.5, p=0.95 | MWU=78, T=169, p=0.35 | MWU=84.5, T=175.5, p=0.95 | X^2^=0.0, p=1 | X^2^=1, p=0.607 |
| **Return latencies after (sec)** |  |  |  |  |  |
| Mounts | MWU=73, T=186, p=0.56 | MWU=73, T=164, p=0.5 | MWU=78.5, T=181.5, p=0.74 | X^2^=3.941, p=0.139 | X^2^=1.514, p=0.469 |
| Intromissions | MWU=65, T=195, p=0.33 | MWU=75, T=166, p=0.7 | MWU=70.5, T=161.5, p=0.48 | X^2^=0.154, p=0.926 | X^2^=1.077, p=0.584 |
| Ejaculations | MWU=81, T=179, p=0.85 | MWU=84, T=175, p=1 | MWU=81.5, T=178.5, p=0.89 | X^2^=4.308, p=0.116 | X^2^=6.292, p=0.043 |
| **Percentage of exits after** |  |  |  |  |  |
| Mounts | MWU=83, T=177, p=0.95 | MWU=71, T=162, p=0.41 | MWU=79, T=181, p=0.763 | X^2^=0.97, p=0.616 | X^2^=389, p=0.823 |
| Intromissions | MWU=71.5, T=188.5, p=0.5 | MWU=58, T=149, p=0.2 | MWU=82.5, T=173.5, p=0.94 | X^2^=12.12, p=0.002 | X^2^=7.385, p=0.025 |
| Ejaculations | MWU=78, T=182, p=0.723 | MWU=78, T=182, p=0.57 | MWU=71.5, T=188.5, p=0.3 | X^2^=6.25, p=0.044 | X^2^=5.25, p=0.072 |
