# Supplementary material for: Brain circuits activated by female sexual behavior evaluated by manganese enhanced magnetic resonance imaging
Source: PLoS One. 2022 Aug 1;17(8):e0272271. doi: 10.1371/journal.pone.0272271 (PMC9342731; doi:10.1371/journal.pone.0272271)
Supplement: S4 Table — a) Statistical results comparing the signal intensity of the different ROIs from 8 mg/kg group in experiment 2 using the Mann-Whitney U test. b) Statistical results comparing the signal intensity of the different ROIs from 16 mg/kg group in experiment 2 using the Mann-Whitney U test. (DOCX) [file pone.0272271.s007.docx]

| **Supplementary Table 4a.** Statistical results comparing the signal intensity of the different ROIs from 8 mg/kg group in experiment 2 using the Mann-Whitney U test. | | | |
| --- | --- | --- | --- |
|  | S1 | S5 | S10 |
| NAcc | MWU=57.5, T=148.5, p=0.174 | MWU=31, T=122, p=0.007 | MWU=37, T=128, p=0.016 |
| AMG | MWU=82.5, T=173.5, p=0.939 | MWU=45, T=136, p=0.045 | MWU=59.5, T=150.5, p=0.209 |
| BNST | MWU=57, T=148, p=0.166 | MWU=22, T=113, p=0.001 | MWU=38.5, T=129.5, p=0.02 |
| Hipp | MWU=54, T=145, p=0.124 | MWU=35.5, T=126.5, p=0.013 | MWU=52, 143, p=1.0 |
| MPOA | MWU=55.5, T=146.5, p=0.144 | MWU=25, T=116, p=0.002 | MWU=37.5, T=128.5, p=0.017 |
| OB | MWU=45, T=136, p=0.045 | MWU=36.5, T=127.5, p=0.015 | MWU=42.5, T=133.5, p=0.033 |
| STR | MWU=58.5, T=149.5, p=0.191 | MWU=29, T=120, p=0.005 | MWU=37.5, T=128.5, p=0.017 |
| VMH | MWU=68, T=159, p=0.412 | MWU=30.5, T=121.5, p=0.006 | MWU=39, T=130, p=0.021 |
| VTA | MWU=62.5, T=153.5, p=0.27 | MWU=41.5, T=132.5, p=0.029 | MWU=38.5, T=129.5, p=0.019 |
|  |  |  |  |
| **Supplementary Table 4b.** Statistical results comparing the signal intensity of the different ROIs from 16 mg/kg group in experiment 2 using the Mann-Whitney U test. | | | |
|  | S1 | S5 | S10 |
| NAcc | MWU=81.5, T=178.5, p=0.898 | MWU=55, T=146, p=0.137 | MWU=44, T=216, p=0.04 |
| AMG | MWU=57, T=203, p=0.166 | MWU=73.5, T=186.5, p=0.59 | MWU=40, T=131, p=0.024 |
| BNST | MWU=82, T=173, p=0.918 | MWU=56.5, T=147.5, p=0.158 | MWU=49, T=140, p=0.072 |
| Hipp | MWU=75, T=185, p=0.644 | MWU=80, T=171, p=0.837 | MWU=54.5, T=145.5, p=0.13 |
| MPOA | MWU=80, T=180, p=0.837 | MWU=54.5, T=145.5, p=0.13 | MWU=40, T=131, p=0.024 |
| OB | MWU=72, T=163, p=0.538 | MWU=58, T=149, p=0.182 | MWU=27, T=118, p=0.003 |
| STR | MWU=78.5, T=181.5, p=0.778 | MWU=59, T150, p=0.2 | MWU=51, T=142, p=0.09 |
| VMH | MWU=63, T=197, p=0.281 | MWU=65, T=156, p=0.33 | MWU=31.5, T=122.5, p=0.007 |
| VTA | MWU=71, T=189, p=0.505 | MWU=82, T=173, p=0.918 | MWU=42, T=133, p=0.031 |
|  | | | |
