# Supplementary material for: Brain circuits activated by female sexual behavior evaluated by manganese enhanced magnetic resonance imaging
Source: PLoS One. 2022 Aug 1;17(8):e0272271. doi: 10.1371/journal.pone.0272271 (PMC9342731; doi:10.1371/journal.pone.0272271)
Supplement: S5 Table — a) Statistical results using the Friedman repeated measures ANOVA comparing the signal intensity in the different ROIs activated by sexual behavior in experiment 2. b). Results from the comparisons within groups using the Kruskal Wallis test on ROIs that activates sexual behavior from experiment 2. c). Statistical results comparing by Friedman repeated measures ANOVA the circuits activated by sexual behavior in experiment 2. (DOCX) [file pone.0272271.s008.docx]

| **Supplementary Table 5a.** Statistical results using the Friedman repeated measures ANOVA comparing the signal intensity in the different ROIs activated by sexual behavior in experiment 2. | | | | | | | |
| --- | --- | --- | --- | --- | --- | --- | --- |
|  | Control 8 mg/kg | | SB 8 mg/kg | | Control 16 mg/kg | | SB 16 mg/kg |
| NAcc | X^2^=1.922, df=2, p=0.383 | | X^2^=1.846, df=2, p=0.397 | | X^2^=0.154, df=2, p=0.926 | | X^2^=10.429, df=2, p=0.005 |
| AMG | X^2^=1.077, df=2, p=0.584 | | X^2^=0.275, df=2, p=0.872 | | X^2^=1.077, df=2, p=0.584 | | X^2^=13, df=2, p=0.002 |
| BNST | X^2^=0.462, df=2, p=0.794 | | X^2^x=1.385, df=2, p=0.5 | | X^2^=0.275, df=2, p=0.972 | | X^2^=10.429, df=2, p=0.005 |
| Hipp | X^2^=0.615, df=2, p=0.735 | | X^2^=1.216, df=2, p=0.545 | | X^2^=0.462, df=2, p=0.794 | | X^2^=9.418, df=2, p=0.009 |
| MPOA | X^2^=0.857, df=2, p=0.651 | | X^2^=0.824, df=2, p=0.662 | | X^2^=0.745, df=2, p=0.689 | | X^2^=11.571, df=2, p=0.003 |
| OB | X^2^=5.087, df=2, p=0.079 | | X^2^=1.385, df=2, p=0.5 | | X^2^=7.569, df=2, p=0.023 | | X^2^=8.143, df=2, p=0.017 |
| STR | X^2^=0.462, df=2, p=0.794 | | X^2^=1.846, df=2, p=0.397 | | X^2^=0.275, df=2, p=0.872 | | X^2^=8, df=2, p=0.018 |
| VMH | X^2^=0.154, df=2, p=0.926 | | X^2^=0.615, df=2, p=0.735 | | X^2^=0.462, df=2, p=0.794 | | X^2^=14.714, df=2, p=<0.001 |
| VTA | X^2^=0.52, df=2, p=0.771 | | X^2^=0.615, df=2, p=0.735 | | X^2^=0.275, df=2, p=0.872 | | X^2^=7.429, df=2, p=0.024 |
|  | | | | | | | |
| **Supplementary Table 5b.** Results from the comparisons within groups using the Kruskal Wallis test on ROIs that activates from sexual behavior from experiment 2 | | | | | | | |
|  | | S1 | | S5 | | S10 | |
| Socio-sexual and Reward circuit | | H=4.032, df=3, p=0.258 | | H=16.811, df=3, p=<0.001 | | H=30.035, df=3, p=<0.001 | |
|  | |  | |  | |  | |
| **Supplementary Table 5c.** Statistical results comparing by Friedman repeated measures ANOVA the circuits activated by sexual behavior in experiment 2. | | | | | | | |
| Control Reward circuit | | SB Reward circuit | | Control Sexual circuit | | SB Sexual circuit | |
| X^2^=0.35, df=2, p=0.840 | | X^2^=37.641, df=2, p=<0.001 | | X^2^=1.298, df=2, p=0.523 | | X^2^=43.857, df=2, p=<0.001 | |
|  | |  | |  | |  | |
|  | | | | | | | |
