# Supplementary material for: Brain circuits activated by female sexual behavior evaluated by manganese enhanced magnetic resonance imaging
Source: PLoS One. 2022 Aug 1;17(8):e0272271. doi: 10.1371/journal.pone.0272271 (PMC9342731; doi:10.1371/journal.pone.0272271)
Supplement: Attachment — Submitted filename: Response to reviewers.docx [file pone.0272271.s009.docx]

Response to reviewers

General

1.- The funding information "This research was supported by grant DGAPA, PAPIIT, UNAM, IN20652. The funders had no role in study design, data collection and analysis, decision to publish, or preparation of the manuscript" is correct and was removed from the Acknowledgments section.

2.- Figures 6 and 11 are now cited in the text. Figures 6 in page 17; Figure 11 in page 21.

3.- The captions for the supplementary figures 1, 2 and 3 are now included at the end of the manuscript.

4.- We checked the reference list

Reviewer 1

1.- We modify the last paragraph of the abstract to clarify that “The socio sexual circuit showed a higher signal intensity on session 5 than the reward circuit and the control groups indicating that even with sexual experience the activation of the reward circuit requires the activation of the socio sexual circuit”

2.- As suggested by the reviewer we added a paragraph in the discussion section about proceptive behaviors and possible long-term effects of MnCl2 on preceptive behaviors: “These measures of the paced mating condition are a better representation of sexual motivation than hops, darts and ear wiggling because the latter ones may or no may occur when females paced the sexual interaction (Erskine 1989) an in some cases they are reduced when females paced in comparison when they don’t pace the sexual interaction (ventura Aquino & Fernandez Guasti. Physiol Behav 120, 70-76; 20139). It is also clear that the administration of MnCl_2_ didn’t modify any of the sexual parameters displayed by the females across the 10 weeks of testing. The MLI and LQ remained high, the percentage of exits and the return latencies are similar to what we (Arzate et al., 2011 H&B 59, 674-680; Camacho et al., 2009 Horm & Behav 56; 410-415; Corona et al., 2011, Horm & Behav 60, 264-268) and others (Erskine 1989) have consistently described.”

3.- We now indicate the doses of the mixture in mg/kg.

4.- In the new version we mentioned at the beginning of experiment 1 that the administration of manganese was subcutaneous.

5.- We modified figures 1,7,8, and supplementary figures 1 and 2 for clarity.

6.- There isn’t much information about MEMRI in other species. In the discussion we described studies in rats, mice, and grasshoppers, but we added a paragraph at the endo of the conclusion to indicate the importance of considering possible toxic effect in other species.

Reviewer 2

As suggested by the reviewer we added a paragraph at the end of the abstract and the conclusion to put in a broader context the usefulness of our study.

Introduction:

1.- As suggested by the reviewer, we now explain the importance of “increasing MR contrast in T1 weighted images”. We added the following paragraph on page 4 “Hence, when the neurons are depolarized, the active cells increase T1 signal intensity by the accumulation of manganese. Therefore, the increase of signal intensity is related to the activation of neurons.

2.- As indicated by the reviewer, we now explicitly mentioned that we investigated female paced sexual behavior

Methods:

1.- As suggested by the reviewer we now include a rationale and references for the EB dose used in the study: “These doses were used to induced high levels of sexual receptivity and we have used them repeatedly before (34, 35)”

2.- As for the number of subjects in experiment, is not necessarily a low number for behavioral studies. In our experience in tests of sexual behavior around 9/10 animals is a good number. We wanted to have 9 subjects per group but in the 8 mg/kg group, one female died of unknown reasons. Another important reason is that the use of the MRI machine is expensive and in experiment 1 we only wanted to find the ideal dose to use in experiment 2 where we could have more animals

3.- Regarding why we didn’t use other measures of proceptivity, is the same response to reviewer 1: we added a paragraph in the discussion section about proceptive behaviors: “These measures of the paced mating condition are a better representation of sexual motivation than hops, darts and ear wiggling because the latter ones may or no may occur when females paced the sexual interaction (Erskine 1989) an in some cases they are reduced when females paced in comparison when they don’t pace the sexual interaction (ventura Aquino & Fernandez Guasti. Physiol Behav 120, 70-76; 20139).

4.- As suggested by the reviewer in the new version we explained how percent of exits and return latencies are obtained, see sexual behavior tests on page 6. “The percentage of exits is obtained by determined the number of times the female exits the male compartment after a mount, intromission, or ejaculation in comparison to the total number of each stimulus received. While the return latencies are calculated by obtaining the mean time for the female to return to the male's chamber after an exit following a mount, an intromission, or an ejaculation”

5.- We modified the abstract to be consistent with the methods section clarifying that in both experiments subjects were injected with MnCl_2_ in sessions 1, 5, and 10: “In experiment 1 we evaluated the effects of two doses of MnCl_2_, 8 and 16 mg/kg. Subjects were injected with one of the doses of MnCl_2_ 24 hours before the test on sessions 1, 5 and 10 and immediately thereafter scanned. Female sexual behavior, running wheel and the rotarod were evaluated once a week for 10 weeks. In experiment 2 we followed a similar procedure, but females……”

6.- The X-axis in Figure 1 is now label “Sessions”

Results:

1.- We appreciate the detailed revision of the discrepancies between what was in the text and the information presented in the supplementary material. We corrected the information in the new version.

2.- As suggested by the reviewer we modified figures 7, 8, and supplementary figures 1 and 2, to have the keys placed in the same graph and modified the legend for clarity.

3.- We also modified pages 17,18 and 19 to make explicit that the significant changes were observed in the SB 16 mg/kg group.

Discussion:

1.- We defined in the discussion serotonin transporter knockout (SERT-KO) mice.

2.- As suggested by both reviewers we added a paragraph at the end of the conclusion to put in a wither perspective the use of MEMRI: “The MEMRI methodology can be use in different species to evaluate in the same animal brain changes associated with a particular behavior, but possible toxic effects should be considered. It is also clear, that we can now evaluate many different aspects of sexual behavior and other motivated behaviors in both males and females, by MEMRI”.
